# Single Aliquot Regeneration (SAR) Optically Stimulated Luminescence Dating Protocols Using Different Grain-Sizes of Quartz: Revisiting the Chronology of Mircea Vodă Loess-Paleosol Master Section (Romania)

**DOI:** 10.3390/mps3010019

**Published:** 2020-02-27

**Authors:** Ștefana-M. Groza-Săcaciu, Cristian Panaiotu, Alida Timar-Gabor

**Affiliations:** 1 Interdisciplinary Research Institute on Bio-Nano-Science, Babes-Bolyai University, 400000 400271 Cluj-Napoca, Romania; smgroza@yahoo.com; 2 Faculty of Environmental Science and Engineering, Babes-Bolyai University, 400000 400294 Cluj-Napoca, Romania; 3 Faculty of Physics, University of Bucharest, 030018 077125 Magurele, Romania; cristian.panaiotu@gmail.com

**Keywords:** luminescence dating, loess, optically stimulated luminescence, single aliquot regeneration protocol, quartz, grain size

## Abstract

The loess-paleosol archive from Mircea Vodă (Romania) represents one of the most studied sections in Europe. We are applying here the current state of the art luminescence dating protocols for revisiting the chronology of this section. Analysis were performed on fine (4–11 µm) and coarse (63–90 µm) quartz extracts using the single aliquot regenerative (SAR) optically stimulated luminescence (OSL) dating protocol. Laboratory generated SAR dose response curves in the high dose range (5 kGy for fine quartz and 2 kGy for coarse quartz) were investigated by employing a test dose of either 17 or 170 Gy. The results confirm the previously reported different saturation characteristics of the two quartz fractions, with no evident dependency of the equivalent dose (D_e_) on the size of the test dose. The OSL SAR ages are discussed and compared to the previously obtained results on quartz and feldspars. The previous reports regarding the chronological discrepancy between the two quartz fractions are confirmed. However, while previous investigations on other sites concluded that this discrepancy appears only above equivalent doses of about 100 Gy, here fine grain quartz ages underestimate coarse quartz ages starting with equivalent doses as low as around 50 Gy.

## 1. Introduction

The development of the single-aliquot regenerative-dose (SAR) protocol [1] for optically stimulated luminescence (OSL) dating of quartz has revolutionized the luminescence dating method by giving rise to high precision equivalent dose estimates. Loess-paleosol sequences are important archives of the climatic changes that took place during the Pleistocene, but their significance can only be fully understood once a reliable and absolute chronology is available. Due to its quartz rich and windblown nature, loess is generally considered an ideal material for the application of OSL. However, although more precise ages can be obtained by SAR-OSL, the validation of the accuracy of these OSL ages by independent age control is hindered by the lack of methods which can directly date the depositional time of the sediments. In this context, the identification of the paleosol associated with marine isotope stage (MIS) 5 is known to yield valuable time control as the identification of this paleosol provides a minimum age threshold for the sediments underlying it, which should be no younger than ~130 ka. However, it is well known that the results of luminescence dating methods applied on quartz underestimate the expected ages for samples collected below this soil. For example, an age of 106 ± 16 ka (equivalent dose of 310 ± 9 Gy) was obtained for quartz grains of 4–11 μm from one sample taken immediately below the S_1_ paleosol (associated with MIS 5) at Mircea Vodă loess paleosol site, Romania, while an increasing degree of age underestimation with depth was observed for samples taken from below S_1_, S_2_ and S_3_ paleosols at the same location [2]. The same trend in age underestimates was reported in China. At Luochuan, Buylaert et al. [3] obtained an age on coarse (63–90 µm) quartz of 81± 7 ka (D_e_= 229 ± 16 Gy) for the loess beneath the S_1_ paleosol while Lai [4] reported lower ages than expected for samples older than 70 ka on 45–63 µm quartz. In the case of another site on the Chinese Loess Plateau (Zhongjiacai), Buylaert et al. [5] obtained for a sample taken from the lower part of the last interglacial paleosol an age of 69.8 ± 3.8 ka (D_e_= 216 ± 6 Gy) on coarse (63–90 µm) quartz. 

Another important issue which was raised relates to the choice of the quartz grain size. The use of coarse grains (so called inclusion dating) or fine grains (4–11 µm) has been proposed five decades ago for thermoluminescence dating of pottery, based on the different penetration powers of nuclear radiations in minerals by Fleming [6] and Zimmerman [7], respectively. However, in later geological applications in what regards OSL dating technique, the choice between these protocols was dictated by the dominant grain size within the investigated sedimentary unit. Consequently, it is common practice to use only one grain size fraction. A series of investigations carried out by our group during the last decade on quartz of different grain sizes extracted from loess yielded intriguing and concerning results. While ages obtained on fine (4–11 μm) and coarse (>63 μm) quartz samples were in good agreement up until ~40 ka, after this age the optical ages obtained on coarse (63–90 μm) quartz were reported to be systematically higher than those on fine (4–11 μm) quartz [8,9,10,11,12,13].

In the light of these findings, we are applying here the single aliquot regeneration dating protocol on quartz of different grain sizes for revisiting the chronology of Mircea-Vodă loess paleosol sequence in Romania. This is the site where we have reported for the first time various problems when investigating different quartz grain sizes [8] and we have subsequently applied alternative luminescence dating protocols on feldspars [14,15]. Additionally, there is a limited practice of performing interlaboratory comparison exercises in the field of luminescence dating. This represents an interesting endeavor since this new investigation takes place a decade later and in different laboratories (Ghent, Belgium [2,8] and Cluj-Napoca, Romania—current paper), using different samples from the same site. We are testing the robustness of the protocol by performing intrinsic rigor tests and we are discussing the accuracy of the obtained ages, also in the light of the results of the previous studies.

## 2. Optically Stimulated Luminescence Dating Methodology

### 2.1. Principles of Luminescence Dating

Optically stimulated luminescence was developed by Huntley et al. [16] and was aimed for establishing the chronology of sediments, especially those where the luminescent signal can be zeroed by exposure to sunlight before deposition such as loess, desert sands and coastal dunes [17]. This dating technique makes use of natural dosimeters (primarily quartz and feldspar grains) that have thermally stable traps capable of storing electrons that arise from the interaction of the environmental ionizing radiation during burial (these radiations coming from the decay of uranium, thorium and potassium in the sediment and from cosmic radiation) with the crystal lattice [18]. This trapped charge population builds up since the time of deposition. As such there is a functionality between the dose received by the crystal (hence the time as the dose rate is assumed to be constant) and the amount of trapped charge. Under controlled laboratory conditions this charge can be quantified in the form of a luminescence signal. The assumption on which the method is based on is that the growth of the luminescence signal in nature can be reproduced by performing controlled laboratory irradiations. Consequently, a dose response curve is constructed and the natural luminescent signal measured in the laboratory is expressed as an equivalent dose by interpolating the natural signal on this dose response curve. The luminescence age equation is shown below. The age is obtained by dividing the equivalent dose value (expressed in Gy) by the dose rate (expressed as Gy/ky).

Age (ky) = Equivalent dose (Gy)/Dose rate (Gy/ky)

### 2.2. Fine (4–11 µm) Versus Coarse (>63 µm) Quartz Grains Dating of Loess

Besides the type of mineral used for OSL measurements, the grain size also plays an important role. Commonly, the size is chosen depending on the dominant grain size of the investigated sedimentary unit. However, it is mandatory to take into consideration the depth at which the alpha, beta and gamma radiation penetrate the grain when age calculation is performed. The silt-sized (4–11 µm) fraction is fully penetrated by all three types of radiations (alpha, beta and gamma, respectively). The sand-sized (>63 µm) grain, on the other hand, receives less of the external beta dose rate due to the attenuation of these radiations in the grain [19,20] and the alpha dose is not homogenously delivered to the grain, the latter being concentrated in an exterior layer which is usually removed by hydrofluoric acid treatment. As a result, the alpha contribution can determine a dose rate for fine grains of even 40% of the total in comparison to coarse grains where the contribution is almost zero [20]. Additionally, an α-efficiency (a-value) must be incorporated when calculating the dose rate for fine grains (~4–11 µm) due to the different efficiency of α-particles compared to β- and γ-radiation in producing luminescence [21]. However, the need for using these different correction factors when dose rates are calculated is well known for decades [20].

It is generally believed that relying only on one fraction for OSL dating should lead to obtaining reliable chronologies. Dating studies on relatively young samples (D_e_ < 100 Gy) using multiple different grain sizes of quartz yielded accurate ages as confirmed by comparison with independent age control provided through tephrochronology [13,22] or radiocarbon dating [23,24]. In this dose range a good agreement has been reported when both fine and coarse quartz were used in order to obtain a chronology of the investigated loess sites [25,26,27]. On the other hand, for older samples the optical ages obtained on coarse quartz (>63–90 µm) were reported to be systematically higher than those on fine quartz (4–11 µm), resulting in a significant difference between the ages obtained on the two grain sizes and raising significant doubts on previously obtained chronologies for ages older than about 50 ka [8,11,12,28]. For large doses (>~500 Gy) the laboratory dose response can be well fitted only by a sum of two single saturating exponential functions [9]. Different saturation characteristics between the fine and coarse quartz fractions extracted from loess were noted, with the fine grains showing higher saturation characteristics worldwide [11]. This is most intriguing when correlated to the fact that the fine fraction underestimates the true ages sooner than the coarse ones. At the moment, the source of the age discrepancy is not fully understood, but it is thought to reside, at least partly, in the different saturation characteristics of fine grains compared to the coarse grains, and in the differences reported between the laboratory and the natural dose response curves as reported by Timar-Gabor and Wintle [29] for Romanian loess as well as by Chapot et al. [30] for loess in China.

## 3. Studied Site

### 3.1. Location and Importance

The Middle and Lower Danube Basins contain the westernmost part of the Eurasian steppe belt, covering the Pannonian Basin and reaching up until the Danube flows into the Black Sea. Here, loess intercalated with paleosols plateaus developed on top of accumulations of fluvial deposits in subsiding areas during the Quaternary. These loess-paleosols sequences (LPSs) are considered to be important and continuous paleoclimatic archives, displaying similar sedimentological and pedological properties to deposits from China and Central Asia [31,32].

**Figure 1:**
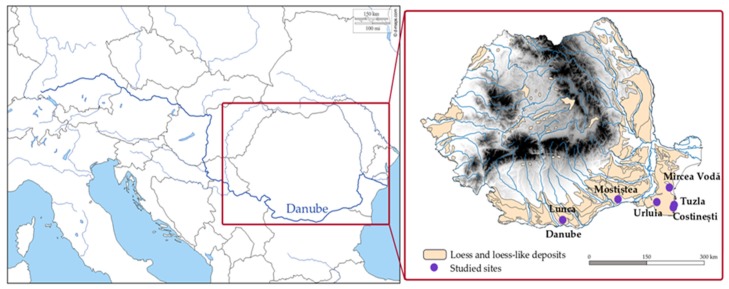
Showing the location of the loess and loess-like deposits in Romania alongside the previously investigated sites—Mircea Vodă, Tuzla, Costinești, Urluia, Mostiștea and Lunca.

The Lower Danube Basin encompasses the area outlined by the Iron Gates gorges, the Black Sea, the Southern Carpathians and the Balkans. The basin is divided into three main regions—the Bulgarian Danube Plain, the Romanian Plain and the Dobrogea Plateau. For the Romanian part, the loess-like deposits are predominant [33]. They were first described in the works of Ana Conea and were given a proposed chronology based on pedostratigraphic methods [34,35]. Dating studies later focused on the Romanian Plain and Dobrogea loess, with a small number of Middle to late Pleistocene loess-paleosol sites being investigated using modern techniques—Mostiștea [14,36,37,38], Lunca [39], Mircea Vodă, Costinești, Tuzla [2,8,12,31,32,35,40,41,42] and Urluia [43] (Figure 1).

The loess-paleosol archive from Mircea Vodă (48°19′15″ N, 28°11′21″ E) is situated in the Dobrogea region, in the proximity of the Danube River, the Black Sea and the Karasu valley. It is considered to be a key section, being one of the most studied sections in Eastern Europe. Six well developed pedocomplexes (covering the last 17 Marine Isotope Stages (MIS)) are comprised in the approximately 26 m thick eolian deposit with no visible hiatuses, overlaying Tertiary and Mesozoic sediments [41].

Previous sedimentological, geochemical and environmental magnetic results showed that the loess from Mircea Vodă displays similarities with the loess from Serbia (Vojvodina) and China (Chinese Loess Plateau) [31,41,44,45,46]. More precisely, the site displays similar major element ratios and geochemical fingerprint as the Serbian loess from the Vojvodina region, with Danube alluvial sediments being also the main loess source [31]. Furthermore, there is a resemblance in the concentration related magnetic parameters, diffuse reflectance spectroscopy results and soil color proxies for hematite and goethite, with the magnetic grain size and mineralogy being also similar to that of Chinese LPSs [44,45,46]. It was observed that the background susceptibilities for Mircea Vodă and Serbian loess sites are in the same range (21 × 10^−8^ −22 × 10^−8^ m^3^·kg^−1^) [31] and that the characteristic magnetic susceptibility patterns of their paleosols can be correlated with corresponding patterns in the susceptibility record of Chinese LPSs [31,41].

Grain-size analysis concluded that throughout the section, silt and fine sand (>16 µm) dominate, while in the lower part of the section there is a larger amount of clay-sized material [8]. The section also exhibits overall pedogenic processes, thus suggesting that loess deposition took place at the same time as weak pedogenesis [8].

From a geochemical point of view, Mircea Vodă exhibits higher carbonate content than the Serbian sites, probably as a result of a more arid climate [31]. The loess units, formed during glacial periods, are dominated by windblown coarse ferromagnetic minerals and have a high quartz content alongside a trend to higher zirconium and hafnium content [31,46]. The paleosol layers are dominated by fine ferromagnetic minerals produced during interglacial pedogenesis, with small amounts of coarser eolian magnetic grains [46]. With the sediment budget being attributed to the Danube River, due to the origin of the quartz and zircon and the bi- and three-modal distribution of the grain-size in loess layers, an additional input from the Ukrainian glaciofluvial deposits and local sand dune fields was also proposed [8,31].

### 3.2. Stratigraphy

As previously mentioned, the Mircea Vodă section displays six pedocomplexes, comprising at least 700 ka of paleoclimate. The S_0_ layer is a steppe soil which displays similarities with the L_3_ unit in what regards magnetic granulometry [31,45]. An interstadial pedocomplex (L_1_S_1_) of the last glacial cycle is comprised in the L_1_ unit [2,31].

The S_1_pedocomplex has been identified as a gray-brown fossil steppe soil [38,41]. It displays three magnetic susceptibility peaks—a dominating peak in the lower half of the unit which may represent MIS 5e and two additional weakly expressed susceptibility peaks, probably representing MIS 5a and MIS 5c [41].

The S_2_pedocomplex has also been identified as a gray-brown fossil steppe soil [38,44]. It comprises three clearly separated peaks and it is attributed to MIS 7. Moreover, due to its characteristic magnetic susceptibility pattern, it can be correlated with the Chinese Loess Plateau sections which show similar enhanced magnetism resulting from interglacial pedogenesis [47].

Due to its paleopedological characteristics, the S_3_ unit can be identified as a fossil steppe or forest-steppe soil [44]. The S_3_ does not show the characteristic double peak like other nearby sections does (Batajnica, Serbia), but it has the strongest magnetic enhancement and it corresponds to MIS 9 [42]. The S_4_ paleosol is correlated with MIS 11 [44].

The S_5_ paleosol is correlated with MIS 13-15 and has been classified as a fossil (chromic) Cambisol and Luvisol [48]. It is the best-developed soil of the Brunhes-chron therefore it may represent a marker horizon in this area [41]. The S_6_ unit shows two susceptibility peaks, the latter most probably indicating the interglacial formation of MIS 17 or MIS 19 [41].

### 3.3. Previous Studies on Mircea Vodă Section

In order to obtain a continuum time-depth model for the Mircea Vodă section, modeling has been employed on the magnetic susceptibility data by Timar et al. [2] by using Match-2.3 software [49] and the stack of 57 globally distributed benthic δ^18^O records as the target curve [50]. Two tie points have been used for the upper part (0 ka) and the bottom (626 ka) of the section [2]. The modeling results, similar to the previously obtained chronostratigraphy by Buggle et al. [41], show that paleosols and loess units correspond to interglacial and glacial periods, therefore being correlated with odd and even marine isotopes, respectively. Moreover, both models assign the weakly developed paleosol embedded in L_1_ to the MIS 3 interstadial, thus disproving the chronology proposed by Conea [34,35].

**Figure 2:**
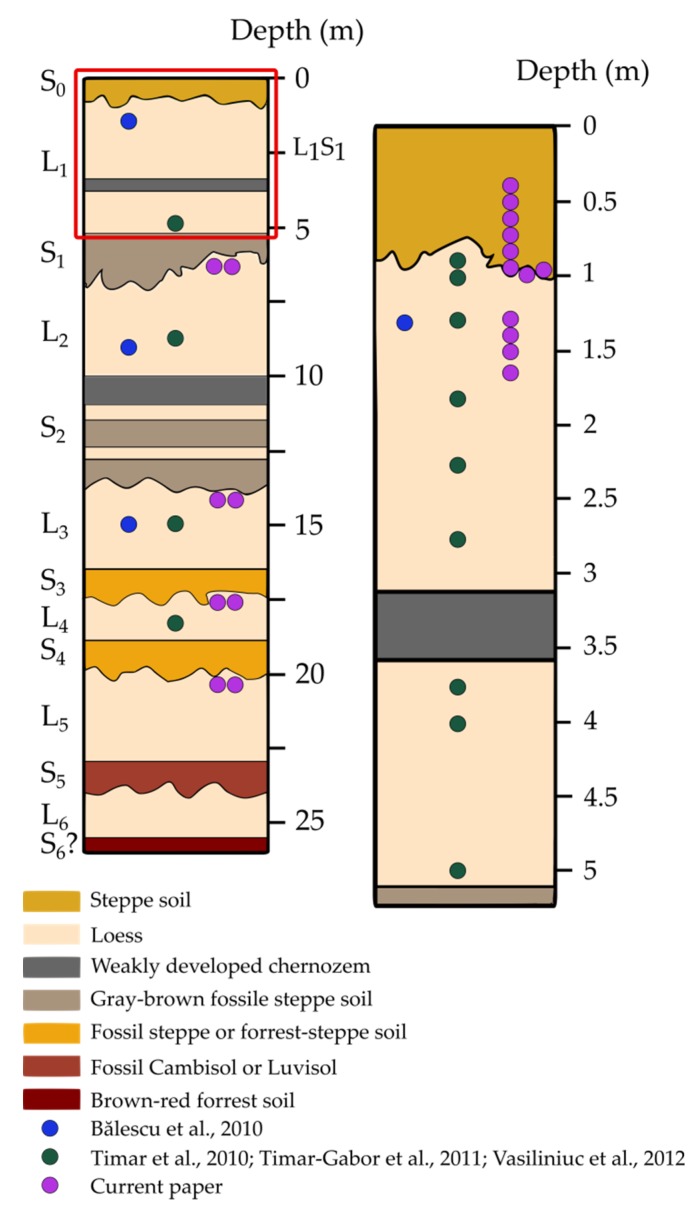
Stratigraphic column of the Mircea Vodă loess-paleosol section (drawn by the authors based on field observation and in accordance with previous stratigraphy presented by a Bălescuet al. [38]). The column on the right represents the first approximately 5 m at a higher resolution. The colored circles represent the position of the samples which were investigated in previous studies and current paper.

Mircea Vodă was the first section in Romania to be dated using optically stimulated luminescence (OSL) methods based on fine quartz (4–11 μm) by Timar et al. [2]. At the same time, Bălescu et al. [38] investigated alkali feldspars extracted from three samples taken from L_1_, L_2_ and L_3_ loess units, with the age results being in broad (stratigraphic interpretation) agreement with those obtained by Timar et al. [2]. The quartz luminescence study of Timar et al. [2] focused on the last four glacial periods, with 9 samples being taken from the uppermost loess layer (L_1_) and three more from L_2_, L_3_ and L_4_ loess units, respectively (Figure 2). Timar-Gabor et al. [8] later presented a comparison on ages obtained on coarse (63–90 μm) quartz. The two OSL datasets were not in agreement as one would generally expect. As a result, the same samples have been investigated by Vasiliniuc et al. [14,15,51] by using polymineral fine (4–11 μm) fraction extracted from the same material used by Timar et al. [2].

#### Luminescence Characteristics and Behavior

The first OSL chronology obtained for the Mircea Vodă section was reported by Timar et al. [2] on fine (4–11 μm) quartz fraction extracted from 12 samples (MV 01–13) (Figure 2). Later on, Timar-Gabor et al. [8] focused on the coarse (63–90 μm) quartz fraction obtained from the same samples. The luminescence characteristics were studied by applying the SAR protocol [1] (Table 1). The OSL signal for both fine (4–11 μm) and coarse (63–90 μm) quartz grains exhibited rapid decay during optical stimulation, with the natural, regenerated and calibration quartz signals being indistinguishable from one another [2,8]. In order to further assess whether the signal is dominated by the fast component, LM-OSL measurements have been performed (Table S1). Once more, the natural signal for both quartz fractions proved to match the signal from the calibration quartz, displaying no significant dependency on the different preheat temperatures used [2]. This was also confirmed by LM-OSL dose response curves (DRC) constructed up to 1 kGy [9].

Moreover, the SAR measurement sequence proved to be accurately corrected for sensitivity changes based on the results obtained for recycling and IR depletion tests (both ratios within 10% from unity). Recuperation tests shown that thermal transfer is not a significant issue either, with signals measured following a zero dose being <0.3% of the sensitivity corrected natural signal. The dose recovery tests (Table S1) also showed that known laboratory given doses can be successfully measured over the entire dose range (from ~28–~480 Gy) for both fine and coarse quartz [2,8].

However, the equivalent doses obtained on fine (4–11 μm) quartz were lower than those obtained on coarse (63–90 μm) quartz, in contradiction with what is presumed considering the expectations based on dose rates [2,8]. In other words, due to the fact that the fine grains have received alpha dose, they should display higher equivalent doses compared to coarse grains. This raised one significant issue—why are different results obtained despite the similar OSL characteristics and behavior? In order to further investigate this issue, pulse annealing measurements on both quartz fractions have been employed in order to assess the potential contamination of the OSL dosimetric trap with an unstable component. The results disproved the contamination scenario, with the signal being confirmed to be thermally stable [8].

The only noticeable difference between the two grain sizes was seen in the response of the signal as function of dose. Sensitivity corrected dose response curves built up to ~700 Gy either fitted with single saturating exponential or a sum of a single saturating exponential and a linear component showed different growth patterns for the two quartz fractions [8] (Table S1). Later on, Timar-Gabor et al. [9] further investigated dose response curves up to 10 kGy for both quartz grain sizes and Timar-Gabor et al. [28] investigated the reproducibility of the dose response curves up to 15 kGy on coarse (63–90 μm) quartz (Table S1). In the first experiment the curves were fitted better with a sum of two saturating exponentials function and the coarse grains saturated much earlier [9]. For the latter experiment the dose response constructed up to 15 kGy could be well reproduced following repeated light exposure and irradiation cycles [28].

The site has also been investigated by Bălescu et al. [38], alongside two more loess sites from Eastern Romania—Tuzla and Mostiștea. At Mircea Vodă, samples have been taken from the L_1_, L_2_ and L_3_ units, from which 60–80 µm alkali feldspars fraction was extracted. The measurement protocol used was the multiple aliquot additive dose method (MAAD) [52] (Table 1).

Bearing in mind the issues rose by the quartz results, Vasiliniuc et al. [14,15,51] tried a different approach. Their studies focused on luminescence properties and ages obtained for polymineral fine (4–11 μm) material extracted from previously investigated samples by Timar et al. [2].

Feldspar dating was carried out by Vasiliniuc et al. [14] who used IRSL signals by employing the post-IR IRSL SAR protocol [5,53] (Table 1). Two preheat post-IR IR stimulation temperature combinations were used. In the first, a 60 s preheat treatment at 250 °C was followed by 100 s IR stimulation at 50 °C (IR_50_) and a second 100 s stimulation at 225 °C (post-IR_50_ IR_225_). The second choice of measurement parameters involved a 60 s preheat treatment at 325 °C was followed by 100 s IR stimulation at 50 °C (IR_50_) and a second 100 s stimulation at 300 °C (post-IR_50_ IR_300_) (Table S1). Residual doses obtained were between 3.8 ± 0.3 Gy and 17.0 ± 0.4 Gy for the post-IR IR_225_ and between 11.3 ± 0.3 Gy and 33.2 ± 1.1 Gy for the post-IR IR_300_, the latter results being similar to those obtained by Thiel et al. [54] and Stevens et al. [55].

The observation of both natural and laboratory induced (during dose recovery tests) signals above the saturation level of the dose response curve, in the case of post-IR IR_300_ signals lead to the conclusion that these signals suffer from dose dependent initial sensitivity changes. On the other hand, the post-IR IR_225_ signals were observed to successfully pass the SAR performance tests in terms of recycling ratio, recuperation and dose recovery. For old samples both natural and regenerated signals (measured during dose recovery tests) were observed to correspond to the saturating region of the dose-response curve, indicating that the small fading rate determined for this signal is probably an artefact of the measurement procedure. The uncorrected ages obtained using the post-IR IR_225_ signals for samples taken from L_2_, L_3_ and L_4_ were found in good agreement with the results of time-depth modelling based on magnetic susceptibility data.

### 3.4. Current Study on Mircea Vodă

#### 3.4.1. Sampling, Preparation and Analytical Facilities

**Figure 3:**
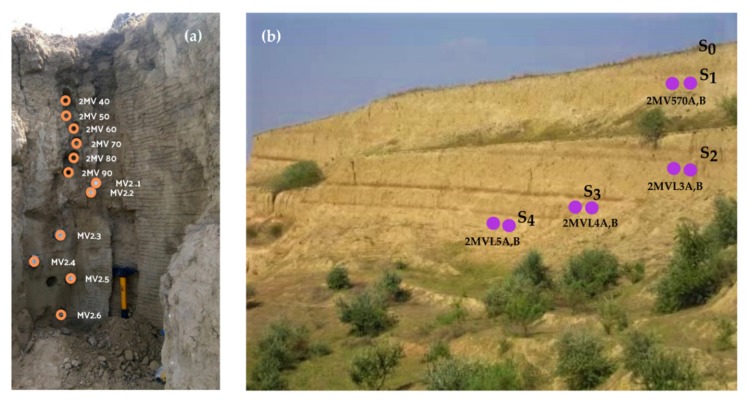
(**a**) Sample positions on the field in the Holocene soil (S_0_) and L_1_ loess unit; (**b**) relative positions for the collected doublet samples beneath the S_1_–S_4_.

For the current paper, investigations were performed on 20 new samples from Mircea Vodă section. The first 12 samples (2MV 40–MV 2.6) were taken from the Pleistocene/Holocene transition (Figure 3a), while doublet samples (2MV 570, L3, L4 and L5) were taken directly beneath the S_1_–S_4_ units, respectively (Figure 3b). The sampling procedure was carried out by using stainless steel tubes inserted horizontally in the freshly cleaned profile.

Standard laboratory sample preparation was then performed under red light conditions. The bulk material was first treated with HCl (35% concentration) for carbonate removal and H_2_O_2_ (30% concentration) for organic matter removal. After each of these steps the samples were rinsed 3 times. The coarse fraction (63–90 μm) was extracted by wet and dry sieving, after which the material was treated with 40% HF for 60 min and a 60 min bath in 10% HCl. Attenberg cylinders were used for obtaining the fine fraction (<11 μm). The material was afterwards etched with 35% hexafluorosilicic acid (H_2_SiF_6_) for 10 days and centrifuged with distilled water in order to attain the 4–11 μm quartz grains [56,57]

For measurement purposes the coarse (63–90 μm) quartz grains were mounted on stainless steel disks using silicone oil as adhesive. The fine (4–11 μm) quartz grains were settled on aluminum disks from a 2 mg/mL suspension in acetone.

The samples were measured on Risø TL/OSL-DA-20 readers [58] with the stimulation being performed by blue light emitting diodes (470 ± 30 nm) and IR light emitting diodes (875 ± 80 nm). The luminescence emissions were detected by an incorporated bialkaline EMI 9235QA photomultiplier (maximum detection efficiency ~400 nm) through a 7.5 mm thick Hoya U-340 UV filter. Irradiations were carried out using a ^90^Sr-^90^Y radioactive source which was calibrated using gamma irradiated fine and coarse calibration quartz [59]. Radionuclide specific activities were measured though high-resolution gamma spectrometry using a coaxial detector with high purity germanium well detector (120 cm^3^ volume), full width at half maximum (FWHM) of 1.40 keV at 122 keV and a full width at half maximum (FWHM) of 2.30 keV at 1332 keV. IAEA 312 and IAEA 327 standards have been used for relative calibration.

#### 3.4.2. Luminescence Measurements

**Figure 4:**
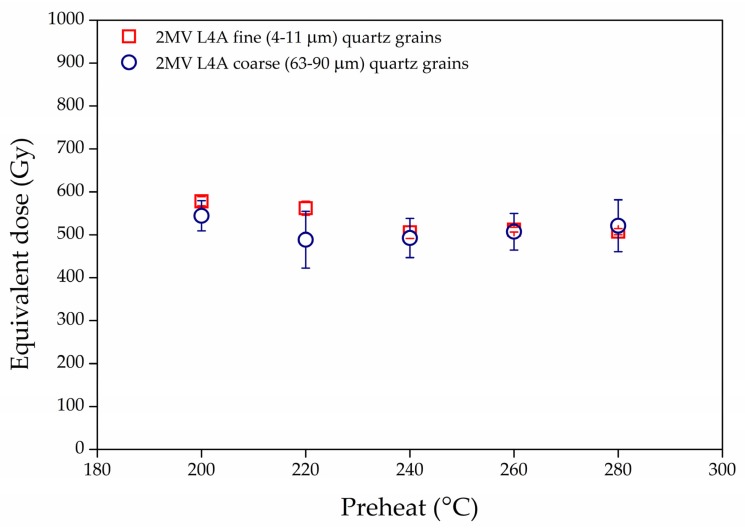
Equivalent dose dependence on preheat temperature for sample 2MV L4A for fine (4–11 µm) quartz fraction (open red squares) and coarse (63–90 µm) quartz fraction (open blue circles).

For **D_e_** determination for the fine (4–11 μm) and coarse (63–90 μm) fractions the single-aliquot regenerative dose (SAR) protocol was used [1,60]. To corroborate the previous quartz studies on Mircea Vodă, the **D_e_** dependency on the preheat treatment was assessed for one doublet sample from the L_4_ unit. The test concluded that there is no systematic variation for the 200–280 °C temperature range (Figure 4). Thus, for consistency reasons with our previous studies a preheat temperature of 220 °C for 10 s and a cutheat of 180 °C have been further used, alongside a test dose of 17 Gy. A high-temperature bleach by stimulation with blue LEDs for 40 s at 280 °C at the end of each test dose signal measurement was employed (Table 1). TL was recorded during the preheat procedure. The OSL signal used was recorded during the first 0.308 s of stimulation and an early background subtraction has been applied from the 1.69–2.31 s interval [61].

**Figure 5:**
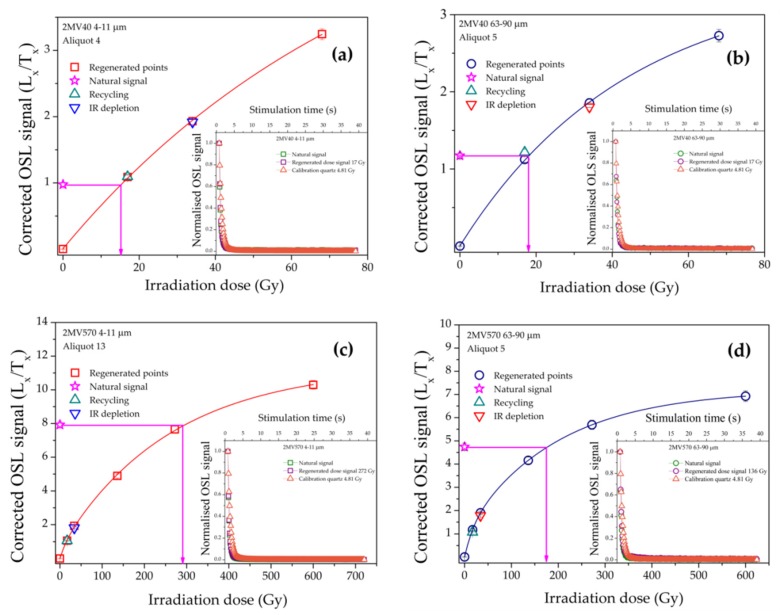
SAR dose response curves for accepted aliquots from samples 2MV40 (**a**, **b**) and 2MV570 (**c**, **d**) for both quartz fractions. Natural signals are represented as stars. Error bars are smaller than the symbols. The comparison between the normalized decay curves (the number of counts in each data channel divided by the number of counts measured in the first channel of stimulation) of the natural OSL signals, the regenerated signals and the decay of the calibration quartz is represented in the insets.

For determining equivalent doses at least 8 aliquots have been measured per sample per quartz fraction. The accepted aliquots exhibited good recycling and IR depletion ratios, though the average values in the case of coarse (63–90 μm) quartz were 2% and 4%, respectively, lower than those obtained by Timar-Gabor et al. [8]. In the case of the 63–90 μm quartz extracts, 22% of the measured aliquots were rejected due to poor IR and recycling, while for the 4–11 μm only 1% of the aliquots were rejected and only due to poor IR depletion values. In Figure 5 representative CW-OSL decay and dose response curves for two samples are presented. By interpolating the sensitivity corrected natural OSL signals onto the dose response curve constructed the equivalent doses were determined (Table 2).

Based on previous reports, the CW-OSL growth curves up to high doses for both fine and coarse quartz is best described by a sum of two saturating exponential functions [65,66,67,68] of the form:

I(D) = I_0_ + A_1_∗(1 − exp(−D/D_01_)) + A_2_∗(1 − exp(−D/D_02_))

where the parameters are: I—intensity of the signal for a given dose D; I_0_—intercept; A_1_, A_2_—saturation amplitudes of the two exponential components; D_01_, D_02_ -doses which represent the onset of saturation of each exponential function.

In order to assess the closeness to saturation of the coarse quartz (63–90 µm) natural signal, CW-OSL growth curves were constructed up to 1 kGy for the old samples taken from L_3_, L_4_ and L_5_ loess units. The ratio between the average sensitivity corrected signal (L_nat_/T_nat_) and the corrected luminescence signals measured for the 1000 Gy regenerative dose (L_x_/T_x1000Gy_) was calculated since for this dose it was observed that the dose response curve is very close to saturation. The coarse (63–90 µm) quartz natural signal for these older samples reached between 71% and 87% of the laboratory saturation level. All equivalent doses are presented in Table 2.

**Figure 6:**
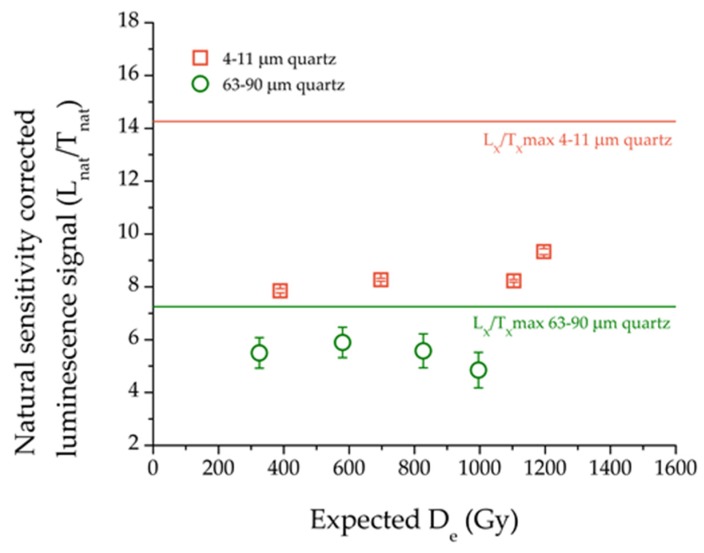
The natural sensitivity corrected luminescence signal (L_x_/T_x_) for samples 2MV570, 2MV3, 2MVL4 and 2MVL5 for 4–11 µm quartz (open squares) and 63–90 µm quartz (open circles) plotted against the expected D_e_. The average maximum sensitivity corrected signal (L_x_/T_x_ max) for a regenerative dose of 2000 Gy in the case of coarse grains and 5000 Gy in the case of fine grains (taken from the data presented in Figure 7) is shown as a reference for both quartz fractions.

**Figure 7:**
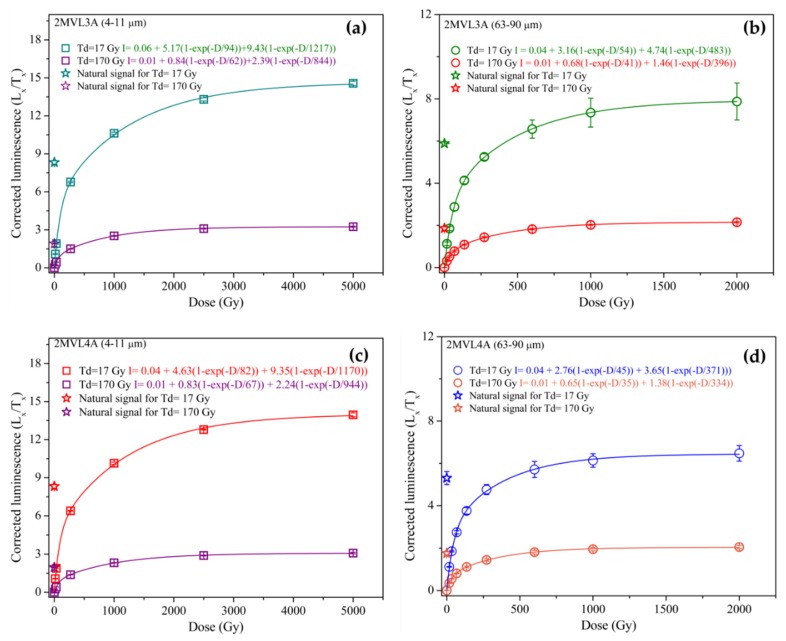
Comparison of growth curves for samples 2MVL3A (**a**,**b**) and 2MVL4A (**c**,**d**) for both quartz fractions. The curves were best described by a sum of two exponential exponentials function. At least three aliquots have been used in order to obtain the average corrected luminescence signals used to construct de growth curves. A preheat temperature of 220 °C for 10 s and a cutheat of 180 °C have been employed.

For both quartz fractions, the average sensitivity-corrected natural signals (L_nat_/T_nat_) for the 2MV570, 2MV3, 2MVL4 and 2MVL5 samples were plotted as a function of the expected D_e_ (Figure 6). Bearing in mind the fact that these samples were taken directly under the paleosol units, the expected ages were found based on the climatic records of benthic δ^18^O [50]. The expected D_e_ was thus calculated by multiplying these ages with the annual dose values obtained in Table 2. A relative uncertainty of 10% was considered. It can be noted that the samples are in field saturation, meaning that the natural signals are no longer increasing with depth. The averaged natural signal for fine quartz is 59% of the average sensitivity corrected signal for the 5000 Gy regenerative dose (a dose high enough for the laboratory dose response to approach saturation—see Figure 7) and the coarse quartz natural signal is 75% of the average sensitivity corrected signal for the 2000 Gy regenerative dose (a dose high enough for the laboratory dose response to approach saturation—see Figure 7). These ratios of the natural signals to laboratory saturation levels for the two grain sizes are in agreement with previous reports from Timar-Gabor et al. [9] for an infinitely old sample. Differences of 60% (4–11 µm quartz) and 80% (63–90 µm quartz) were reported between the natural and laboratory dose response curves constructed for the nearby loess-paleosol site of Costinești [28]. Similar discrepancies between the natural and the laboratory dose response curves were reported by Chapot et al. [30] on samples taken from Luochuan, China.

Extended CW-OSL growth curves were subsequently constructed for samples 2MV L3A and 2MV L4A up to 5 kGy (for 4–11 µm quartz grains) and 2 kGy (for 63–90 µm quartz grains) using at least 6 regenerative points and a test dose of 17 Gy as well as a test dose of 170 Gy (Figure 7a–d). The data was fitted using a sum of two exponential functions (Table 3). Results obtained using a test dose of 17 Gy confirm the different saturation characteristics between the two quartz fractions [9,11,22,28], with average value of the two samples obtained for the fine (4–11 µm) fraction (D_01_ = 88 ± 22 Gy; D_02_= 1194 ± 142 Gy) similar to those reported by Timar-Gabor et al. [11] (D_01_ = 151 ± 5 Gy; D_02_ = 1411 ± 64 Gy) and the coarse (63–90 µm) fraction (D_01_ = 50 ± 8 Gy; D_02_ = 427 ± 54 Gy) close to values from Timar-Gabor et al. [11] (D_01_ = 44 Gy; D_02_ = 452 Gy), Murray et al. [65] (D_01_ = 44 Gy; D_02_ = 450 Gy for 180–250 µm quartz) and Pawley et al. [66] (D_01_ = 51 Gy; D_02_ = 320 Gy for 125–180 µm quartz). For a test dose of 170 Gy, there is a general trend for the D_01_ and D_02_ values to decrease (Figure 7a–d). For the 2MV L3A and 2MV L4A fine (4–11 µm) quartz the D_01_ values decreased by 34% and 18%, while the D_02_ values decreased by 31% and 19%, respectively. The D_01_ values for coarse (63–90 µm) quartz were lower than those using a test dose of 17 Gy by 24% and 22% and the D_02_ values were lower by 18% and 10%, respectively (Table 3). As a result, the use of the 170 Gy test dose increased the closeness of the natural corrected luminescence signals to the saturation levels for both quartz fractions by 3.5% up to 16%, with the coarse (63–90 µm) quartz for the two oldest samples reaching ≥86% of saturation level. The equivalent doses were obtained by measuring at least 3 aliquots (Table 3). Even though there is a slight increase in the values obtained the results remain consistent within uncertainties. It should be noted that poor recycling ratios have been observed for fine (4–11 µm) quartz when the 170 Gy test dose was employed (4 aliquots measured), with values lower than unity by 27%. However, this effect was not significant for the coarse (63–90 µm) quartz measured with a test dose of 170 Gy with recycling values contained in the 0.9–1.1 interval.

## 4. Ages and Discussion

**Figure 8:**
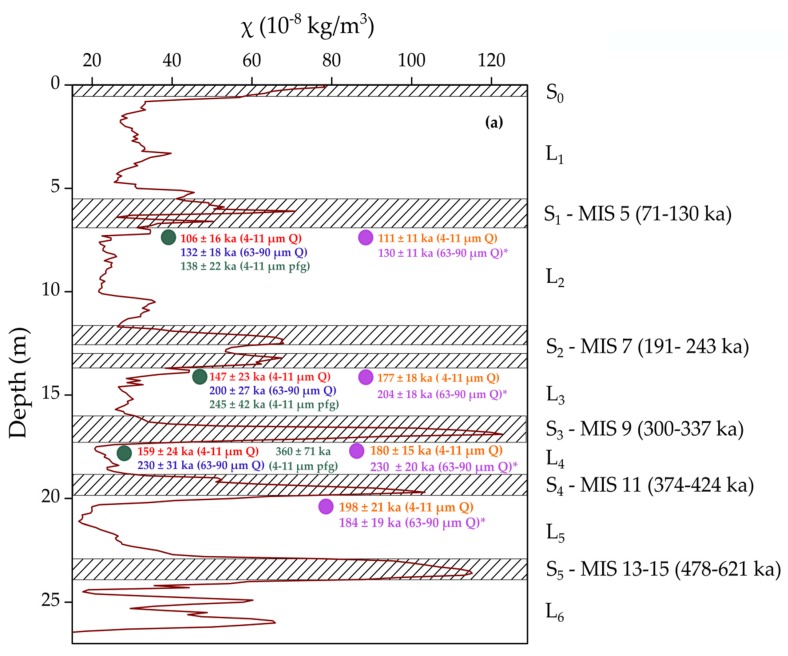

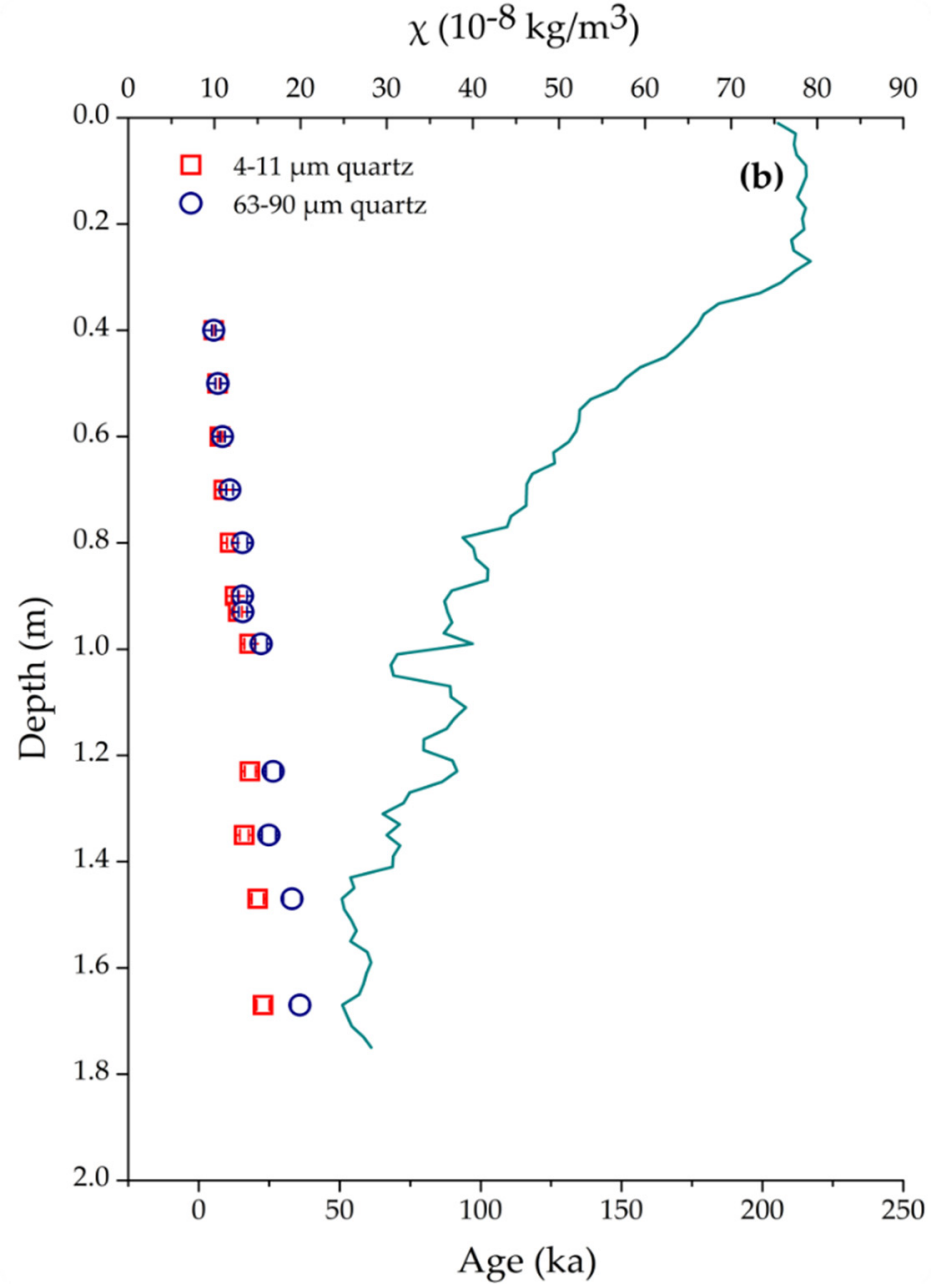
(**a**) Schematic representation of the loess (L) and paleosol units (S; hatched area) with magnetic susceptibility values (χ) from [2].The boundaries of paleosols developed during odd marine isotope stages (MIS) are after [49]. The ages for the old samples (green circles) and new samples (purple circles) are shown as follows: written in red—[2]; blue—[8]; green—[14] and written in orange—4–11 μm quartz current paper; purple—63–90 µm quartz current paper. The current ages represent the weighted results from the doublet samples. The optical ages marked with asterisk (*) were obtained for samples which were found to be close to saturation levels. (**b**) Plot of new optical ages as a function of depth alongside new magnetic susceptibility data. The fine (4–11 µm) quartz ages are represented as open squares and the coarse (63–90 µm) quartz ages are represented as open circles.

Previous luminescence dating studies on Mircea Vodă site (Figure 8a) revealed an age discrepancy between the two quartz fractions investigated that is still not yet understood. The ages obtained ranged from 8.7 ± 1.3 to 159 ± 24 ka for fine silt-sized (4–11 μm) quartz and from 16 ± 2 ka to 230 ± 31 ka for fine sand-sized (63–90 μm) quartz, the difference varying between 20% to 70% [2,8]. Despite the fact that both datasets were consistent with the stratigraphic position of the samples, the fine (4–11 μm) quartz ages for the three samples taken from the L_2_, L_3_ and L_4_ loess units were interpreted as underestimates. The post-IR IR_225_ signal was considered more reliable than the previously obtained quartz ages for the L_2_, L_3_ and L_4_ units. These ages are presented alongside the quartz ages in Figure 8a.

Due to the importance of Mircea Vodă loess-paleosol master section, the current study aimed to obtain a more detailed chronological framework. In this regard 13 samples have been collected from the Holocene soil (S_0_) and L_1_ loess unit and doublet samples have been collected directly beneath the S_1_–S_4_ units, respectively. The luminescence ages were found to be mostly in agreement with their stratigraphically corresponding results reported by Timar-Gabor et al. [8] and Vasiliniuc et al. [14]. The overall uncertainties associated with the new OSL ages are dominated by the systematic uncertainties caused by the time-averaged water content, a-value and beta attenuation factors. The overall contribution from random sources of uncertainties scatters around 2.5% for the fine (4–11 μm) quartz (generally 10 aliquots per sample) and ranges from 4.2% to 11.8% for the coarse (63–90 μm) quartz. It has been proposed that the source for such a large spread could be attributed to reduced number of coarse quartz grains on the disk compared to fine aliquots, thus reducing the variability in the luminescence properties. Other studies have also reported similar spread in coarse quartz data [23,25].

The top part of the profile that encompasses the Pleistocene/Holocene transition (L_1_/S_0_ unit) is characterized by increasing ages with profile depth, ranging from 5.3 ± 0.5 ka to 22.8 ± 2.3 ka for fine (4–11 μm) quartz and from 5.3 ± 0.7 ka to 35.9 ± 3.2 ka for coarse (63–90 μm) quartz (Figure 8b). In the Holocene soil, for ages up to about 11 ka OSL ages obtained for coarse and fine quartz agree, as expected and previously reported for such young samples [27]. The older samples taken from the L_1_/S_0_ transition and the L_1_ loess unit yielded ages that no longer agree within uncertainties. The fine (4–11 μm) ages continue to be younger than coarse (63–90 μm) ages. As previously reported by Timar-Gabor et al. [8] on Mircea Vodă, Timar-Gabor et al. [9] on Mostiștea, Constantin et al. [12] on Costinești, Timar-Gabor et al. [10] on Orlovat in Serbia as well as by Timar-Gabor et al. [11] in China, the equivalent doses are higher for coarse (63–90 μm) quartz, which is unexpected considering the annual dose rate.

The age discrepancy between the two quartz fractions appears to begin sooner than previously reported. For Romanian, Serbian and Chinese loess samples, SAR-OSL ages divergence arose beyond ~40 ka (>~100 Gy) [11]. Only for samples collected from Lunca section (southern Wallachian Plain, see Figure 1) such a difference occurred starting with samples as young as <30 ka (~80 Gy) [39].

For the samples starting with L_2_ downwards, the results obtained on doublet samples on each quartz fractions were found in agreement within uncertainties. Consequently, weighted ages have been calculated for each grain size fraction on the doublet samples.

The doublet samples collected from the L_2_ loess unit yielded weighted ages on the two quartz fractions of 111 ± 11 ka for fine (4–11 μm) quartz and 130 ± 11 ka for coarse (63–90 μm) quartz. Fine quartz ages underestimate the expected age, while the coarse quartz age is in broad agreement with the expected age. These results are consistent with those reported by Timar-Gabor et al. [8] and Vasiliniuc et al. [14].

The weighted average ages recovered from the L_3_ samples were that of 177 ± 18 ka for 4–11 μm quartz and 204 ± 18 ka for 63–90 μm quartz. The fine quartz age clearly underestimates the expected age, the offset being of 37%, while for the coarse quartz age the underestimation is of 19%. In the case of coarse quartz it was observed that the signal was close to laboratory saturation levels, with an average of 80%, while in the case of fine quartz, the natural signals were interpolated significantly below the saturation level of the laboratory dose response curve.

In what regards the samples from L_4_ and L_5_ loess units, the ages on fine (4–11 μm) quartz are 180 ± 15 ka and 198 ± 21 ka and the ages for coarse (63–90 μm) quartz are 230 ± 20 ka and 184 ± 19 ka, respectively. The two set of ages underestimate severely the expected values from stratigraphical boundaries considerations [50]. The coarse(63–90 μm) quartz signals were found to be close to laboratory saturation for both L_4_ and L_5_ samples (85% and 77%, respectively), with the closeness to laboratory saturation being more pronounced when a larger test dose was used. On the other hand, the same statement cannot be made for fine (4–11 μm) quartz (e.g., for 2MVL5 sample the natural signals were at 60% of the laboratory saturation level).

The reasons behind the age discrepancies between fine (4–11 μm) quartz and coarse (63–90 μm), especially in the case of the Mircea Vodă site have been proposed, discussed and investigated in previous studies. Microdosimetry could be a reason, but one should bear in mind the fact that such an age discrepancy could only arise from a dose rate difference of approximately 1 Gy/ka [8]. The purity of the quartz extracts was checked by comparing the natural and regenerated signals with the calibration quartz, by performing the IR depletion test [69] and by checking the 110 °C TL peak recorder during preheats. The results dismiss the possibility of a feldspathic component which would end in different age results between the two fractions. This also concurs with previous time resolved OSL (TR-OSL) results reported by Timar-Gabor et al. [28]. In what regards partial bleaching, residual doses of the order of at least tens or even hundreds of Gy would be needed in order to cause such an age offset for all samples investigated. This is not to be expected in the case of quartz.

## 5. Conclusions

Fine (4–11 μm) and coarse (63–90 μm) quartz have been investigated by applying SAR-OSL protocol in order to augment the existing chronological framework from Mircea Vodă loess-paleosol master section. The age results for the Pleistocene/Holocene transition have shown that fine and coarse fractions agree only up to ~20 ka. For samples older than this, fine grains quartz ages underestimate coarse quartz ages. Thus, the discrepancy between two datasets occurs sooner than previously shown for other sites [11]. The reason for this difference is yet not understood.

As previously reported the 63–90 μm quartz does not underestimate the expected geological ages and agrees with post-IR IR_225_ for samples collected just below S_1_. For older samples coarse quartz SAR OSL signals approach (86%) laboratory saturation and also enter field saturation. For the counterpart fine grains quartz OSL natural signals are significantly below laboratory saturation levels. However, the fine quartz ages underestimate the expected ages. Therefore, these ages should be taken as minimum ages. Investigation on extended growth curves up to 5 kGy (for 4–11 µm quartz grains) and 2 kGy (for 63–90 µm quartz grains) using test doses of a different order of magnitude (17 and 170 Gy) have concluded that the equivalent dose was insensitive to the size of the test dose.

## Figures and Tables

**Table 1:** Previous studies and protocols used for the chronology of Mircea Vodă section.

Authors/Year	Mineral	Stratigraphical Units Investigated	Measurement Protocol
Bălescu, S.; Lamothe, M.; Panaiotu, C.; Panaiotu, C. (2010) [38]	alkali feldspars (60–80 µm)	1 sample from L_2_ 1 sample from L_3_ 1 sample from L_4_	Multiple aliquot additive dose method (MAAD)
Timar, A.; Vandenberghe, D.; Panaiotu, E.C.; Panaiotu, C.G.; Necula, C.; Cosma, C.; van den haute, P. (2010) [2]	quartz (4–11 µm)	9 samples from L_1_ 1 sample from L_2_ 1 sample from L_3_ 1 sample from L_4_	SAR (CW-OSL)
Timar-Gabor, A.; Vandenberghe, D.A.G.; Vasiliniuc, Ș.; Panaiotu, E.C.; Panaiotu, C.G.; Dimofte, D.; Cosma, C. (2011) [8]	quartz (63–90 µm)	9 samples from L_1_ 1 sample from L_2_ 1 sample from L_3_ 1 sample from L_4_	SAR (CW-OSL)
Timar-Gabor, A.; Vasiliniuc, S.; Vandenberghe, D.A.G.; Cosma, C.; Wintle, A.G.; (2012) [14]	quartz (4–11 and 63–90 µm)	2 samples from L_1_ 1 sample from L_2_ 1 sample from L_3_	SAR (CW-OSL) Dose response curves constructed up to 1200 Gy using LM-OSL signals
Vasiliniuc, Ș.; Vandenberghe, D.A.G.; Timar-Gabor, A.; Panaiotu, C.; Cosma, C.; van den Haute, P. (2012) [14]	polymineral grains (4–11 µm)	5 samples from L_1_ 1 sample from L_2_ 1 sample from L_3_ 1 sample from L_4_	Post- IR IR_225, 300_
Vasiliniuc, Ș.;Vandenberghe, D.A.G.;Timar-Gabor, A.;Cosma, C.; Van Den haute, P. (2013) [51]	polymineral grains (4–11 µm)	9 samples from L_1_ 1 sample from L_2_ 1 sample from L_3_ 1 sample from L_4_	Double SAR (CW-OSL)
Vasiliniuc, Ș.;Vandenberghe, D.A.G.;Timar-Gabor, A.; van den Haute, P. (2013) [15]	polymineral grains (4–11 µm)	9 samples from L_1_ 1 sample from L_2_ 1 sample from L_3_ 1 sample from L_4_	Modified SAR -IRSL at 115°C and 250°C
Timar-Gabor, A.; Constantin, D.; Buylaert, J.P.; Jain, M.; Murray, A.S.; Wintle, A.G. (2015) [28]	quartz (63–90 µm)	2 samples from L_1_	SAR (CW-OSL) Dose response curves constructed up to 15 kGy

**Table 2:** Summary of the equivalent doses, radionuclide activities, calculated dose rates and optical ages. The luminescence and dosimetry data are indicated alongside the random uncertainties and the optical ages are indicated alongside the overall uncertainties. All uncertainties are standard uncertainties. Specific activities were measured on a well detector by high resolution gamma spectrometry. The ages were calculated assuming water content of 20%. The total dose rate includes the contribution from cosmic rays [62], gamma, beta and alpha (for 4–11 µm quartz grains) radiations. An internal dose rate of 0.01 ± 0.002 Gy/ka [63] alongside a beta attenuation and etching factor of 0.94 ± 0.05 [19] were taken into consideration for the coarse (63–90 µm) quartz fraction. The alpha efficiency factor for the 4–11 µm quartz grains was that of 0.04 ± 0.02 [64]. The optical ages marked with asterisk (*) were obtained for samples which were found to be close to saturation levels (between 71% and 87%, Figure 6).

Unit Code	Sampling Depth (m)	Laboratory Code	Grain Size (µm)	Equivalent Dose (Gy)	Recycling Ratio	Recuperation (%)	IR depletion Ratio	Total dose Rate (Gy/ka)	Cosmic dose Rate (Gy/ka)	Age (ka)	Random Error (%)	Systematic Error (%)
S_0_/L_1_	0.4	2MV 40	4–11	15.4 ± 0.2	1.02 ± 0.01	0.11 ± 0.03	0.99 ± 0.01	2.91 ± 0.05	0.22 ± 0.03	5.3 ± 0.5	2.3	9.9
			63–90	12.9 ± 1.5	1.03 ± 0.01	0.15 ± 0.07	0.98 ± 0.01	3.44 ± 0.05		5.3 ± 0.7	11.8	7.6
S_0_/L_1_	0.5	2MV 50	4–11	20.3 ± 0.3	1.02 ± 0.01	0.07 ± 0.03	0.95 ± 0.01	3.05 ± 0.07	0.21 ± 0.03	6.7 ± 0.7	2.7	9.9
			63–90	17.3 ± 1.9	1.05 ± 0.01	0.14 ± 0.04	0.98 ± 0.01	2.55 ± 0.06		6.8 ± 0.9	11.2	7.6
S_0_/L_1_	0.6	2MV 60	4–11	23.6 ± 0.5	1.04 ± 0.01	0.05 ± 0.05	0.99 ± 0.01	3.15 ± 0.05	0.21 ± 0.03	7.5 ± 0.8	2.7	9.9
			63–90	22.1 ± 1.6	1.02 ± 0.01	0.21 ± 0.10	0.98 ± 0.01	2.64 ± 0.05		8.4 ± 0.9	7.4	7.6
S_0_/L_1_	0.7	2MV 70	4–11	25.6 ± 0.3	1.03 ± 0.01	0.07 ± 0.03	0.98 ± 0.01	2.88 ± 0.05	0.20 ± 0.03	8.9 ± 0.9	2.1	9.9
			63–90	26.6 ± 2.1	1.03 ± 0.01	0.09 ± 0.02	0.98 ± 0.01	2.41 ± 0.05		11.0 ± 1.2	8.1	7.6
S_0_/L_1_	0.8	2MV 80	4–11	31.2 ± 0.4	1.00 ± 0.01	0.07 ± 0.03	0.95 ± 0.01	2.81 ± 0.05	0.20 ± 0.03	11.1 ± 1.1	2.0	10.0
			63–90	36.4 ± 2.9	1.02 ± 0.01	0.09 ± 0.03	0.95 ± 0.01	2.35 ± 0.05		15.5 ± 1.7	8.2	7.6
S_0_/L_1_	0.9	2MV 90	4–11	35.9 ± 0.6	0.99 ± 0.01	0.19 ± 0.04	0.98 ± 0.01	2.77 ± 0.05	0.19 ± 0.03	12.9 ± 1.3	2.5	10.0
			63–90	36.0 ± 2.5	1.03 ± 0.01	0.05 ± 0.02	0.97 ± 0.01	2.32 ± 0.05		15.5 ± 1.6	7.2	7.6
S_0_/L_1_	0.93	MV 2.1	4–11	28.3 ± 0.6	1.04 ± 0.01	0.03 ± 0.03	0.94 ± 0.01	2.75 ± 0.04	0.19 ± 0.03	14.0 ± 1.4	2.3	9.7
			63–90	36.1 ± 2.0	1.02 ± 0.01	0.11 ± 0.03	0.98 ± 0.01	2.31 ± 0.04		15.6 ± 1.5	5.8	7.6
S_0_/L_1_	0.99	MV 2.2	4–11	51.7 ± 0.6	0.99 ± 0.01	0.04 ± 0.01	0.98 ± 0.01	2.88 ± 0.05	0.19 ± 0.03	18.0 ± 1.8	2.2	9.7
			63–90	53.6 ± 2.6	1.01 ± 0.01	0.04 ± 0.02	0.97 ± 0.01	2.42 ± 0.05		22.1 ± 2.1	5.2	7.7
S_0_/L_1_	1.23	MV 2.3	4–11	51.2 ± 0.9	1.02 ± 0.02	0.04 ± 0.02	0.90 ± 0.01	2.81 ± 0.06	0.18 ± 0.03	18.2 ± 1.9	2.8	9.9
			63–90	62.1 ± 3.0	1.01 ± 0.01	0.15 ± 0.07	0.97 ± 0.01	2.53 ± 0.05		26.4 ± 2.5	5.3	7.6
S_0_/L_1_	1.35	MV 2.4	4–11	46.8 ± 0.8	1.03 ± 0.02	0.04 ± 0.02	0.98 ± 0.01	2.92 ± 0.04	0.18 ± 0.03	16.1 ± 1.6	2.2	9.9
			63–90	60.8 ± 3.8	1.02 ± 0.01	0.09 ± 0.03	0.97 ± 0.01	2.44 ± 0.04		24.9 ± 2.5	6.4	7.7
S_0_/L_1_	1.47	MV 2.5	4–11	59.7 ± 1.0	1.03 ± 0.01	0.04 ± 0.02	0.92 ± 0.01	2.85 ± 0.05	0.18 ± 0.03	20.9 ± 2.1	2.3	9.9
			63–90	79.0 ± 4.1	1.00 ± 0.01	0.06 ± 0.02	0.97 ± 0.01	2.39 ± 0.04		33.1 ± 3.1	5.5	7.7
S_0_/L_1_	1.67	MV 2.6	4–11	67.4 ± 0.6	1.00 ± 0.01	0.05 ± 0.01	0.98 ± 0.01	2.91 ± 0.05	0.17 ± 0.03	22.8 ± 2.3	2.0	10.1
			63–90	86.9 ± 3.5	1.00 ± 0.01	0.06 ± 0.03	0.98 ± 0.01	2.42 ± 0.04		35.9 ± 3.2	4.4	7.7
L_2_	5.70	2MV 570A	4–11	331 ± 6	0.96 ± 0.01	0.10 ± 0.01	0.95 ± 0.01	3.03 ± 0.06	0.11 ± 0.02	109 ± 11	2.6	10.0
			63–90	303 ± 13	0.97 ± 0.01	0.06 ± 0.02	0.96 ± 0.01	2.54 ± 0.05		120 ± 11*	4.7	7.8
L_2_	5.70	2MV 570B	4–11	331 ± 3	0.96 ± 0.01	0.09 ± 0.01	0.96 ± 0.01	2.95 ± 0.06	0.11 ± 0.02	112 ± 12	2.2	10.1
			63–90	353 ± 13	0.97 ± 0.01	0.05 ± 0.02	0.95 ± 0.01	2.46 ± 0.05		143 ± 13*	4.2	7.8
L_3_	13.70	2MV L3A	4–11	514 ± 16	0.98 ± 0.01	0.06 ± 0.01	0.98 ± 0.01	2.88 ± 0.05	0.06 ± 0.01	179 ± 19	2.4	10.3
			63–90	475 ± 19	0.96 ± 0.01	0.010 ± 0.02	0.94 ± 0.01	2.39 ± 0.04		199 ± 18*	4.3	8.0
L_3_	13.70	2MV L3B	4–11	501 ± 11	0.98 ± 0.01	0.05 ± 0.01	0.99 ± 0.01	2.86 ± 0.06	0.06 ± 0.01	175 ± 18	2.2	10.2
			63–90	501 ± 23	0.95 ± 0.01	0.11 ± 0.02	0.94 ± 0.01	2.38 ± 0.05		210 ± 20*	5.0	8.0
L_4_	17.70	2MV L4A	4–11	567 ± 9	0.99 ± 0.01	0.05 ± 0.002	1.00 ± 0.01	3.14 ± 0.05	0.04 ± 0.01	180 ± 19	2.3	10.4
			63–90	577 ± 23	0.97 ± 0.01	0.13 ± 0.02	0.97 ± 0.01	2.60 ± 0.05		222 ± 20*	-	-
L_4_	17.70	2MV L4B	4–11	477 ± 6	1.00 ± 0.01	0.07 ± 0.004	0.91 ± 0.01	3.41 ± 0.06	0.04 ± 0.01	140 ± 13	2.2	8.8
			63–90	555 ± 26	0.98 ± 0.01	0.17 ± 0.03	0.95 ± 0.01	2.31 ± 0.05		240 ± 23*	-	-
L_5_	20.50	2MV L5A	4–11	566 ± 8	0.98 ± 0.01	0.07 ± 0.003	0.98 ± 0.01	2.66 ± 0.06	0.04 ± 0.01	213 ± 22	2.5	10.2
			63–90	425 ± 39	0.98 ± 0.01	0.21 ± 0.09	0.98 ± 0.01	2.22 ± 0.05		192 ± 24*	-	8.0
L_5_	20.50	2MV L5B	4–11	556 ± 13	0.99 ± 0.01	0.06 ± 0.01	0.98 ± 0.01	2.98 ± 0.06	0.04 ± 0.01	187 ± 20	2.9	-
			63–90	443 ± 35	0.94 ± 0.02	0.43 ± 0.10	0.97 ± 0.03	2.48 ± 0.05		179 ± 20*	8.1	8.0

**Table 3:** **Fitting** parameters for OSL dose response curves constructed up to 5000 Gy (4–11 µm quartz) and 2000 Gy (63–90 µm quartz) for samples 2MV L3A and 2MV L4A.

**Sample**	**Test Dose (Gy)**	**y_0_**	**y_0_Error**	**A_1_**	**A_1_ Error**	**D_01_**	**D_01_Error**	**A_2_**	**A_2_ Error**	**D_02_**	**D_02_Error**	**Reduced χ^2^**	**R^2^**	**D_e_ (Gy)**	**Closeness of Natural Signal to Laboratory Saturation Level (%)**
4–11 μm 2MV L3A	17	0.06	0.14	5.2	0.4	94	18	9.4	0.4	1217	112	0.022	0.999	493 ± 28	57
	170	0.01	0.02	0.8	0.06	62	8	2.4	0.06	844	42	0.05 × 10^−2^	0.999	505 ± 26	59
4–11 μm 2MV L4A	17	0.04	0.12	4.6	0.3	82	13	9.4	0.3	1170	88	0.017	0.999	584 ± 29	60
	170	0.01	0.03	0.8	0.08	67	12	2.2	0.07	944	63	0.09 × 10^−2^	0.999	604 ± 47	63
**Sample**	**Test Dose (Gy)**	**y_0_**	**y_0_ Error**	**A_1_**	**A_1_ Error**	**D_01_**	**D_01_ Error**	**A_2_**	**A_2_ Error**	**D_02_**	**D_02_ Error**	**Reduced χ^2^**	**R^2^**	**D_e_ (Gy)**	
63–90 μm 2MV L3A	17	0.04	0.05	3.2	0.2	54	4	4.7	0.2	483	30	0.002	0.999	584 ± 29	74
	170	0.01	0.02	0.7	0.05	41	5	1.5	0.05	396	25	0.03 × 10^−2^	0.999	560 ± 73	86
63–90 μm 2MV L4A	17	0.04	0.07	2.8	0.3	45	6	3.7	0.2	371	41	0.005	0.999	440 ± 27	83
	170	0.01	0.02	0.7	0.06	35	5	1.4	0.05	334	24	0.3 × 10^−2^	0.999	628 ±89	89

## Supplementary Materials

The following are available online at https://www.mdpi.com/2409-9279/3/1/19/s1.
