# Supplementary material for: Single Aliquot Regeneration (SAR) Optically Stimulated Luminescence Dating Protocols Using Different Grain-Sizes of Quartz: Revisiting the Chronology of Mircea Vodă Loess-Paleosol Master Section (Romania)
Source: Methods Protoc. 2020 Feb 27;3(1):19. doi: 10.3390/mps3010019 (PMC7189666; doi:10.3390/mps3010019)
Supplement: Supplementary file 1 [file mps-03-00019-s001.pdf]

Authors/Year	Mineral used for luminescence measurements	Measurement protocol used to obtain the loess/paleosol ages	Intrinsic rigor tests
Bălescu, S., Lamothe, M., Panaiotu, C., Panaiotu, C. (2010)	alkali feldspars (60-80μm)	<u>Multiple aliquot additive dose method (MAAD)</u><br>o γ irradiation at room temperature<br>o Preheat at 160°C for 10 hours<br>o Measurement (8 to 12 months after γ irradiation)<br>o Preheat at 220°C for 10 minutes<br>o IR stimulation for 30 s<br>o Beta irradiation<br>o Preheat at 220°C for 10 minutes<br>o IR stimulation for 30 s	
Timar,A., Vandenberghe, D., Panaiotu, E.C., Panaiotu, C.G., Necula, C., Cosma, C., van den haute, P., (2010)	quartz (4-11 μm)	<u>SAR (CW-OSL)</u><br>o Preheat at 220°C for 10s<br>o OSL at 125°C for 40s<br>o Test dose (17.1 Gy)<br>o Cutheat at 180°C for 0s<br>o OSL at 125°C for 40s<br>o OSL at 280°C for 40s<br>o Beta irradiation<br><br>Net signal<br>o Initial 0.32s of the decay curve<br>o Background integrated between 1.6 and 2.24s<br><br>Water content assumed to be 20 ± 5%<br>α value adopted: 0.04 ± 0.02<br>Contribution of cosmic rays: Prescott and Hutton, 1994<br>Fitting equation: single saturation exponential plus a linear term	<u>OSL signal assessment - SAR (LM-OSL)</u><br>o Preheat at 220°C for 10s<br>o OSL at 125°C from 0 to 100% in 3000s<br>o Test dose (17.1 Gy)<br>o Cutheat at 180°C for 0s<br>o OSL at 125°C from 0 to 100% in 3000s<br>o OSL at 280°C for 40s<br>o Beta irradiation<br><br><u>Dependency of ED on the preheat temperature (CW-OSL)</u><br>o 200-280°C<br><br><u>Dose recovery test (CW-OSL)</u><br>o Using different preheat temperatures (dose used – 420 Gy)<br>o Using a preheat of 220°C and a cutheat of 180°C

Timar-Gabor, A., Vandenberghe, D.A.G., Vasiliniuc, Ş., Panaiotu, E.C., Panaiotu, C.G., Dimofte, D., Cosma, C. (2011)	quartz (63-90 µm)	<u>SAR (CW-OSL)</u><br>o Preheat at 220°C for 10s<br>o OSL at 125°C for 40s<br>o Test dose (17.1 Gy)<br>o Cutheat at 180°C for 0s<br>o OSL at 125°C for 40s<br>o OSL at 280°C for 40s<br>o Beta irradiation<br><br>Net signal<br>o Initial 0.32s of the decay curve<br>o Background integrated between 1.6 and 2.24s<br><br>Water content assumed to be 20 ± 5%<br>Beta attenuation factor: 0.94 ± 0.045<br>Internal dose rate: 0.010 ± 0.002 Gy/ka (Vandenberghe et al., 2008)<br>Fitting equation: single saturation exponential plus a linear term<br>Contribution of cosmic rays: Prescott and Hutton, 1994	<u>Dependency of ED on the preheat temperature (LM-OSL)</u><br>o Preheat (180-340°C) for 2 s<br><br><u>Dose recovery test (CW-OSL)</u><br>o Using a preheat of 220°C and a cutheat of 180°C<br><br><u>Thermal stability - pulse annealing (CW-OSL)</u><br>o OSL at 20°C for 250s<br>o 10ks pause<br>o OSL at 20°C for 250s<br>o Beta irradiation (D=ED)<br>o Preheat (175-450°C) for 10s<br>o OSL at 125°C for 40s<br>o Test dose (17 Gy)<br>o Cutheat at 180°C for 0s<br>o OSL at 125°C for 40s<br>o OSL at 280°C for 40s<br><br><u>Dose response (CW-OSL)</u><br>o Preheat at 220°C for 10s<br>o OSL at 125°C for 40s<br>o Test dose (17 Gy)<br>o Cutheat at 180°C for 0s<br>o OSL at 125°C for 40s<br>o OSL at 280°C for 40s<br>o Beta irradiation up to ~700 Gy<br><br>Net signal<br>o Initial 0.32s of the decay curve<br>o Background integrated between 1.6 and 2.24s<br><br>Fitting equations: single saturation exponential plus a linear term and a single saturating exponential

Timar-Gabor, A., Vasiliniuc, S., Vandenberghe, D.A.G., Cosma, C., Wintle, A.G., (2012)	quartz (4-11 and 63-90 μm)		Dose response (CW-OSL)<br>o Preheat at 220°C for 10s<br>o OSL at 125°C for 40s<br>o Test dose (17 Gy)<br>o Cutheat at 180°C for 0s<br>o OSL at 125°C for 40s<br>o OSL at 280°C for 40s<br>o Beta irradiation up to 10kGy<br><br>Net signal<br>o Initial 0.3s of the decay curve<br>o Background integrated between 2.3 and 3.1s<br><br>Fitting equations: single saturation exponential or a double saturating exponential
Vasiliniuc, Ș., Vandenberghe, D.A.G., Timar-Gabor, A., Panaiotu, C., Cosma, C., van den Haute, P. (2012)	polymineral grains (4-11 μm)	Modified SAR (CW-OSL)<br>o Dose<br>o Preheat at 250°C or 325°C for 60s<br>o IRSL at 50°C for 100s<br>o IRSL at 225°C or 300°C for 100s<br>o Test dose (10 Gy)<br>o Preheat at 250°C or 325°C for 60s<br>o IRSL at 50°C for 100s<br>o IRSL at 225°C or 300°C for 100s<br>o IRSL at 290°C or 340°C for 40s<br><br>Water content assumed to be 20 ± 5%<br>Mean α-value: 0.08 ± 0.002<br>Fitting equation: single saturation exponential plus a linear term and sum of two saturating exponential functions	Dose response<br>o Doses up to 4 and 12 ka<br><br>Dose recovery – modified SAR (CW-OSL)<br>o 1h bleach in Hönle SOL2 solar simulator<br>o 1h pause<br>o Dose (first one close to the equivalent dose obtained)<br>o Preheat at 250°C or 325°C for 60s<br>o IRSL at 50°C for 100s<br>o IRSL at 225°C or 300°C for 100s<br>o Test dose (10 Gy)<br>o Preheat at 250°C or 325°C for 60s<br>o IRSL at 50°C for 100s<br>o IRSL at 225°C or 300°C for 100s<br>o IRSL at 290°C or 340°C for 40s<br><br>Fading test<br>o IR bleach at 50°C for 100s<br>o IRSL at 250°C or 325°C for 100s

			o 1h pause<br>o IR bleach at 50°C for 100s<br>o IRSL at 250°C or 325°C for 100s<br>o Dose (50 Gy)<br>o Preheat at 250°C or 325°C for 60s<br>o IRSL at 50°C for 0.2h to few tens of hours<br>o IRSL at 225°C or 300°C for 0.2h to few tens of hours<br>o Test dose (20 Gy)<br>o Preheat at 250°C or 325°C for 60s<br>o IRSL at 50°C for 100s<br>o IRSL at 225°C or 300°C for 100s<br>o IRSL at 290°C or 340°C for 40s<br><br>g-value calculated using Huntley and Lamothe (2001); normalized to a measurement delay time of 2 days after irradiation
Vasiliniuc, Ş., Vandenberghe, D.A.G., Timar-Gabor, A., Cosma, C., Van Den haute, P. (2013a)	polymineral grains (4-11 μm)	<u>Double SAR (CW-OSL)</u><br>o Dose<br>o Preheat at 240°C for 10s<br>o IRSL at 125°C for 100s<br>o OSL at 125°C for 100s<br>o Test dose (10 Gy)<br>o Cutheat at 180°C for 0s<br>o IRSL at 125°C for 100s<br>o OSL at 125°C for 100s<br>o OSL at 280°C for 40s<br><br>Water content assumed to be 20 ± 5%<br>Mean α-value: 0.08 ± 0.002 and 0.06 ± 0.02<br>Fitting equation: single saturation exponential function and sum of two saturating exponential functions	<u>Dose recovery - double SAR (CW-OSL)</u><br>o 1h bleach in Hönle SOL2 solar simulator, followed by 1h pause. Preheat at 240°C and at different temperatures (200-260°C) for 10s.<br><br><u>Dependency of D_e on the preheat temperature - double SAR (CW-OSL)</u><br>o Preheat (200-260°C) for 10s<br><br>Fading test<br>o IRSL bleach at 125°C for 250s<br>o OSL bleach at 125°C for 250s<br>o 1h pause<br>o IRSL bleach at 125°C for 250s<br>o OSL bleach at 125°C for 250s<br>o Dose (25 or 50 Gy)<br>o Preheat at 240°C for 10s<br>o IRSL at 125°C for 0.2h to few tens of

			hours<br>o OSL at 125°C for 0.2h to few tens of hours<br>o Test dose (10 or 25 Gy)<br>o Cutheat at 180°C for 0s<br>o IRSL at 125°C for 100s<br>o OSL at 125°C for 100s<br>o OSL at 280°C for 40s<br><br>g-value calculated using Huntley and Lamothe (2001); normalized to a measurement delay time of 2 days after irradiation
Vasiliniuc, Ș., Vandenberghe, D.A.G., Timar-Gabor, A., van den Haute, P. (2013b)	polymineral grains (4-11 μm)	<u>Modified SAR (CW-OSL)</u><br>o Dose<br>o Preheat at 250°C for 60s<br>o IRSL at 115°C or 250°C for 100s<br>o Test dose (10 Gy)<br>o Preheat at 250°C for 60s<br>o IRSL at 115°C or 250°C for 100s<br>o IRSL at 290°C for 40s<br><br>Water content assumed to be 20 ± 5%<br>Mean α-value: 0.08 ± 0.002<br>Fitting equation: sum of two saturating exponential functions<br><br>Net signal<br>o Initial 1.2s of the decay curve<br>o Background from the last 10s	<u>Dose recovery - modified SAR (CW-OSL)</u><br><br>➢ Samples bleached in the solar simulator<br>o 1h bleach in Hönle SOL2 solar simulator<br>o 1h pause<br>o Dose (close to the equivalent dose obtained or close to the natural dose using a preheat of 250°C for 60s)<br>o Preheat at different temperatures (80-325°C) for 60s<br>o IRSL at 115°C or 250°C for 100s<br>o Test dose (10 Gy)<br>o Preheat (80-325° C) for 60s<br>o IRSL at 115°C or 250°C for 100s<br>o IRSL at 290°C for 40s<br>➢ Samples bleached using infrared light<br>o IRSL bleach at 20°C for 250s<br>o IRSL bleach at 20°C for 250s<br>o 1h pause<br>o IRSL bleach at 20°C for 250s<br>o IRSL bleach at 20°C for 250s<br>o Dose (close to the equivalent dose

obtained or close to the natural dose using a preheat of 250°C for 60s)

- o Preheat (80-325° C) for 60s
- o IRSL at 115°C or 250°C for 100s
- o Test dose (10 Gy)
- o Preheat (80-325° C) for 60s
- o IRSL at 115°C or 250°C for 100s
- o IRSL at 290°C for 40s

➢ Unbleached samples

- o Dose (500 Gy on top of natural)
- o Preheat at 115°C or 250°C for 60s
- o IRSL at 115°C or 250°C for 100s
- o Test dose (10 Gy)
- o Preheat at 115°C or 250°C for 60s
- o IRSL at 115°C or 250°C for 100s
- o IRSL at 290°C for 40s

➢ Dose recovery as a function of given dose

- o 1h bleach in Hönle SOL2 solar simulator or 4h unfiltered sunlight
- o Dose
- o Preheat at 250°C for 60s
- o IRSL at 115°C or 250°C for 100s
- o Test dose (10 Gy)
- o Preheat at 250°C for 60s
- o IRSL at 115°C or 250°C for 100s
- o IRSL at 290°C for 40s

<u>Dependency of D_e on the preheat temperature</u>

- o Preheat at different temperatures (80-325°C) for 60s

<u>Fading test</u>

- o IRSL at 115°C or 250°C for 100s
- o 1h pause
- o IRSL at 115°C or 250°C for 100s

			o Dose (25 Gy)<br>o Preheat at 250°C for 60s<br>o IRSL at 225°C or 300°C for 0.14h to ~21h<br>o Test dose (20 Gy)<br>o Preheat at 250°C for 60s<br>o IRSL at 115°C or 250°C for 100s<br>o IRSL at 290°C or 340°C for 40s<br>g-value calculated using Huntley and Lamothe (2001); normalized to a measurement delay time of 2 days after irradiation<br><br><u>Thermal stability</u><br>o Thermoluminescence (TL) glow curves<br>o Multiple-aliquot pulse annealing
Timar-Gabor, A., Constantin, D., Buylaert, J.P., Jain, M., Murray, A.S., Wintle, A.G., (2015)	quartz (63-90 μm)		<u>Dose response (CW-OSL)</u><br>o Preheat at 220°C for 10s<br>o OSL at 125°C for 40s<br>o Test dose (17 Gy)<br>o Cutheat at 180°C for 0s<br>o OSL at 125°C for 40s<br>o OSL at 280°C for 40s<br>o Beta irradiations up to 15kGy<br>o Preheat at 220°C for 10s<br>o OSL at 125°C for 40s<br>o Test dose (17 Gy)<br>o Cutheat at 180°C for 0s<br>o OSL at 125°C for 40s<br>o OSL at 280°C for 40s<br>o OSL bleach at 20°C for 100 s<br>o Pause for 10 000 s<br>o OSL bleach at 20°C for 100 s<br>o Beta irradiation of 170 Gy<br>o OSL at 125°C for 40s

- Test dose (17 Gy)
- Cutheat at 180°C for 0s
- OSL at 125°C for 40s
- OSL at 280°C for 40s
- Beta irradiations up to 15kGy
- Preheat at 220°C for 10s
- OSL at 125°C for 40s
- Test dose (17 Gy)
- Cutheat at 180°C for 0s
- OSL at 125°C for 40s
- OSL at 280°C for 40s
- OSL bleach at 20°C for 100 s

Net signal

- Initial 0.3s of the decay curve
- Background integrated between 2.3 and 3.1s

Fitting equation: double saturating exponential

1. Bălescu, S.; Lamothe, M.; Panaiotu, C.; Panaiotu, C. La chronologie IRSL des séquences loessiques de l'est de la Roumanie. *Quaternaire,* **2010**, *21*, pp. 115-126, DOI: 10.4000/quaternaire.5488.
2. Huntley, D.J.; Lamothe, M. Ubiquity of anomalous fading in k-feldspars and the measurement and correction for it in optical dating. *Can. J. Earth Sci..*, **2001**, *38*, pp. 1093-1106
3. Precott, J.R.; Hutton, J.T. Cosmic ray contributions to dose rates for luminescence and ESR dating: large depths and long-term time variations. *Radiat. Meas.* **1994**, *23*, pp. 497-500, DOI:10.1016/1350-4487(94)90086-8.
4. Timar, A.; Vandenberghe, D.; Panaiotu, E.C.; Panaiotu, C.G.; Necula, C.; Cosma, C.; van den haute, P. Optical dating of Romanian loess using fine-grained quartz. *Quat. Geochronol.* 2010, *5*, pp. 143-148, DOI: 10.1016/j.quageo.2009.03.003.
5. Timar-Gabor, A.; Constantin, D.; Buylaert, J.P.; Jain, M.; Murray, A.S.; Wintle, A.G. Fundamental investigations of natural and laboratory generated SAR dose response curves for quartz OSL in the high dose range. *Radiat. Meas.* **2015**, *81*, pp. 150-156, DOI: 10.1016/j.radmeas.2015.01.013.
6. Timar-Gabor, A.; Vandenberghe, D.A.G.; Vasiliniuc, S.; Panaiotu, C.E.; Panaiotu, C.G.; Dimofte, D.; Cosma, C. Optical dating of Romanian loess: A comparison between silt-sized and sand-sized quartz. *Quat. Int.* **2011**, *240*, pp. 62-70, DOI: 10.1016/j.quaint.2010.10.007.

7. Timar-Gabor, A.; Vasiliniuc, Ș.; Vandenberghe, D.A.G.; Cosma, C.; Wintle, A.G. Investigations into the reliability of SAR-OSL equivalent doses obtained for quartz samples displaying dose response curves with more than one component. *Radiat. Meas.* **2012**, *47*, pp. 740-745, DOI: 10.1016/j.radmeas.2011.12.001.
8. Vangenberghe, D.; De Corte, F.; Buylaert, J.-P.; Kučera, J.; Van den haute, P. On the internal radioactivity of quartz. *Radiat. Meas.* **2008**, *43*, pp. 771-775, DOI:10.1016/j.radmeas.2008.01.016.
9. Vasiliniuc, Ș.; Vandenberghe, D.A.G.; Timar-Gabor, A.; Cosma, C.; van den Haute, P. Combined IRSL and post-IR OSL dating of Romanian loess using single aliquots of polymineral fine grains. *Quat. Int.* **2013a**, *293*, pp. 15-21, DOI: 10.1016/j.quaint.2012.01.002.
10. Vasiliniuc, Ș.; Vandenberghe, D.A.G.; Timar-Gabor, A.; Panaiotu, C.; Cosma, C.; van den Haute, P. Testing the potential of elevated temperature post-IR IRSL signals for dating Romanian loess. *Quat. Geochronol.* **2012**, *10*, pp. 75-80, DOI: 10.1016/j.quageo.2012.02.014.
11. Vasiliniuc, S.; Vandenberghe, D.A.G.; Timar-Gabor, A.; Van Den haute, P. Conventional IRSL dating of Romanian loess using single aliquots of polymineral fine grains. *Radiat. Meas.* **2013b**, *48*, pp. 60-67, DOI: 10.1016/j.radmeas.2012.11.008.
